# 4-Ethynyl-2,2,6,6-tetra­methyl-1,2,5,6-tetra­hydro­pyridine *N*-oxide

**DOI:** 10.1107/S1600536809004681

**Published:** 2009-02-13

**Authors:** Jan W. Bats, Olga Frolow, Joachim W. Engels

**Affiliations:** a Institut für Organische Chemie, Universität Frankfurt, Max-von-Laue-Strasse 7, D-60438 Frankfurt am Main, Germany

## Abstract

The six-membered ring of the title compound, C_11_H_16_NO, has a distorted envelope conformation. The piperidine N atom deviates by 0.128 (1) Å from the plane through its three neighbouring atoms. In the crystal structure, mol­ecules are connected by inter­molecular C_ethyn­yl_—H⋯O contacts to form chains extending in the [10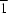] direction.

## Related literature

For the preparation of the title compound, see: Gannett *et al.* (2001 ▶); Frolow *et al.* (2007 ▶). For the crystal structures of related compounds see: Igonin *et al.* (1990 ▶); Wiley *et al.* (1991 ▶); Shklover *et al.* (1990 ▶).
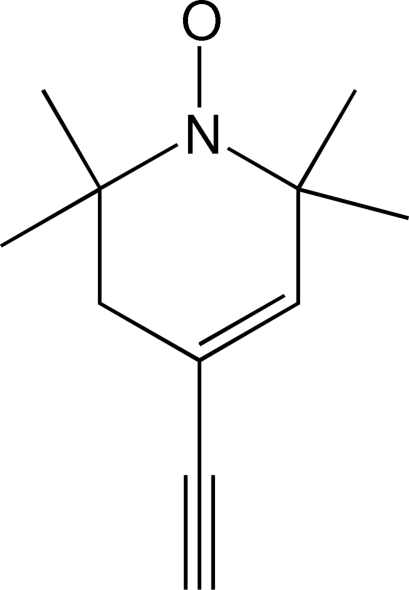


## Experimental

### 

#### Crystal data

C_11_H_16_NO
*M* *_r_* = 178.25
Monoclinic, 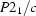

*a* = 6.0996 (9) Å
*b* = 20.800 (3) Å
*c* = 8.3662 (13) Å
β = 97.434 (10)°
*V* = 1052.5 (3) Å^3^
*Z* = 4
Mo *K*α radiation
μ = 0.07 mm^−1^
*T* = 167 K
0.60 × 0.50 × 0.50 mm

#### Data collection

Siemens SMART 1K CCD diffractometer
Absorption correction: none
18416 measured reflections
3580 independent reflections
3143 reflections with *I* > 2σ(*I*)
*R*_int_ = 0.039

#### Refinement

*R*[*F* ^2^ > 2σ(*F* ^2^)] = 0.040
*wR*(*F* ^2^) = 0.112
*S* = 1.09
3580 reflections
131 parameters
H atoms treated by a mixture of independent and constrained refinement
Δρ_max_ = 0.34 e Å^−3^
Δρ_min_ = −0.17 e Å^−3^

### 

Data collection: *SMART* (Siemens, 1995 ▶); cell refinement: *SMART*; data reduction: *SAINT* (Siemens, 1995 ▶); program(s) used to solve structure: *SHELXS97* (Sheldrick, 2008 ▶); program(s) used to refine structure: *SHELXL97* (Sheldrick, 2008 ▶); molecular graphics: *SHELXTL* (Sheldrick, 2008 ▶); software used to prepare material for publication: *SHELXL97*.

## Supplementary Material

Crystal structure: contains datablocks global, I. DOI: 10.1107/S1600536809004681/su2096sup1.cif

Structure factors: contains datablocks I. DOI: 10.1107/S1600536809004681/su2096Isup2.hkl

Additional supplementary materials:  crystallographic information; 3D view; checkCIF report

## Figures and Tables

**Table 1:** Hydrogen-bond geometry (Å, °)

*D*—H⋯*A*	*D*—H	H⋯*A*	*D*⋯*A*	*D*—H⋯*A*
C7—H7*A*⋯O1^i^	0.944 (14)	2.354 (15)	3.2318 (13)	154.6 (13)

Symmetry code: (i) 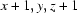.

**Fig. 1.**
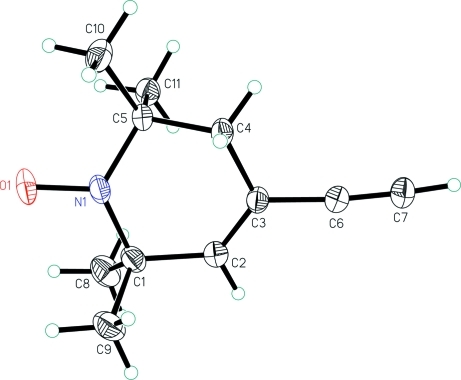
The molecular structure of the title compound, shown with 50% probability displacement ellipsoids. H atoms are drawn as small spheres of arbitrary radiius.

**Fig. 2.**
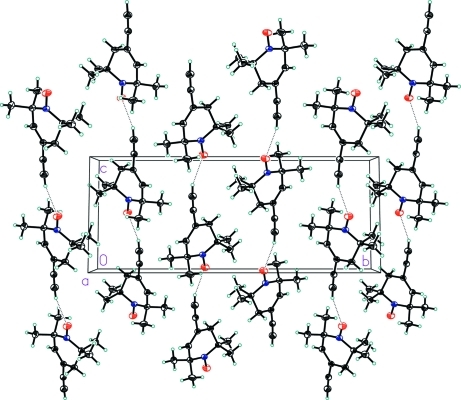
The crystal packing of the title compound, viewed down the a axis. Intermolecular Cethynyl—H···O contacts are shown as dashed lines.

## supplementary crystallographic information

### Comment

For EPR measurements of RNA, DNA or proteins, the occurrence of paramagnetic species is required. The title compound is a nitroxide spin label compound. Its synthesis and application for DNA labeling have been reported by Gannett *et al.* (2001). Frolow *et al.* (2007) reported an improved synthesis of the compound and its coupling to uridine. Here we report on the crystal structure of the title compound.

The molecular structure of the title compound is shown in Fig. 1. The geometrical parameters in the title compound are very similar to those in the 2,2,6,6-tetramethyl-1-oxyl-3,4-dehydropiperidine fragment of closely related molecules (Igonin *et al.*, 1990; Wiley *et al.*, 1991; Shklover *et al.*, 1990). The six-membered ring has a distorted envelope conformation with atoms N1 and C5 deviating by 0.186 (1) and 0.725 (2) Å, respectively, in the same direction from the mean plane through atoms C1-C4 [planar to within 0.005 (1) Å]. Atom N1 shows a small degree of pyramidalization. The sum of the three valence angles about N1 is 357.6 (1)° and it deviates by 0.128 (1) Å from the plane through the three neighbouring atoms, O1, C1 and C5.

In the crystal structure molecules are connected by intermolecular C_ethynyl_—H···O contacts to form chains extending in the [1 0 -1] direction (Fig. 2 and Table 1).

### Experimental

The synthesis of the title compound has been reported by Frolow *et al.* (2007). Crystals were obtained by sublimation at atmospheric pressure.

### Refinement

The H atoms at C2 and C7 were located in difference Fourier maps and freely refined: C-H = 0.973 (13) and 0.944 (15) Å, respectively. The remainder of the H atoms were positioned geometrically and treated as riding: C-H = 0.98 - 0.99 Å with *U*_iso_H = k × *U*_eq_(C), where k = 1.2 for (CH and CH_2_) and 1.5 for (CH_3_).

### Crystal data

**Table:**

C_11_H_16_NO	*F*(000) = 388
*M**_r_* = 178.25	*D*_x_ = 1.125 Mg m^−^^3^
Monoclinic, *P*2_1_/*c*	Mo *K*α radiation, λ = 0.71073 Å
Hall symbol: -P 2ybc	Cell parameters from 212 reflections
*a* = 6.0996 (9) Å	θ = 3–23°
*b* = 20.800 (3) Å	µ = 0.07 mm^−^^1^
*c* = 8.3662 (13) Å	*T* = 167 K
β = 97.434 (10)°	Block, yellow
*V* = 1052.5 (3) Å^3^	0.6 × 0.5 × 0.5 mm
*Z* = 4	

### Data collection

**Table:**

Siemens SMART 1K CCD diffractometer	3143 reflections with *I* > 2σ(*I*)
Radiation source: normal-focus sealed tube	*R*_int_ = 0.039
graphite	θ_max_ = 32.2°, θ_min_ = 2.0°
ω scans	*h* = −8→8
18416 measured reflections	*k* = −31→27
3580 independent reflections	*l* = −12→12

### Refinement

**Table:**

Refinement on *F*^2^	Secondary atom site location: difference Fourier map
Least-squares matrix: full	Hydrogen site location: inferred from neighbouring sites
*R*[*F*^2^ > 2σ(*F*^2^)] = 0.040	H atoms treated by a mixture of independent and constrained refinement
*wR*(*F*^2^) = 0.112	*w* = 1/[σ^2^(*F*_o_^2^) + (0.05*P*)^2^ + 0.2*P*] where *P* = (*F*_o_^2^ + 2*F*_c_^2^)/3
*S* = 1.09	(Δ/σ)_max_ = 0.002
3580 reflections	Δρ_max_ = 0.34 e Å^−^^3^
131 parameters	Δρ_min_ = −0.17 e Å^−^^3^
0 restraints	Extinction correction: *SHELXL97* (Sheldrick, 2008), Fc^*^=kFc[1+0.001xFc^2^λ^3^/sin(2θ)]^-1/4^
Primary atom site location: structure-invariant direct methods	Extinction coefficient: 0.074 (6)

### Special details

**Table:**

Geometry. All e.s.d.'s (except the e.s.d. in the dihedral angle between two l.s. planes) are estimated using the full covariance matrix. The cell e.s.d.'s are taken into account individually in the estimation of e.s.d.'s in distances, angles and torsion angles; correlations between e.s.d.'s in cell parameters are only used when they are defined by crystal symmetry. An approximate (isotropic) treatment of cell e.s.d.'s is used for estimating e.s.d.'s involving l.s. planes.
Refinement. Refinement of *F*^2^ against ALL reflections. The weighted *R*-factor *wR* and goodness of fit *S* are based on *F*^2^, conventional *R*-factors *R* are based on *F*, with *F* set to zero for negative *F*^2^. The threshold expression of *F*^2^ > σ(*F*^2^) is used only for calculating *R*-factors(gt) *etc*. and is not relevant to the choice of reflections for refinement. *R*-factors based on *F*^2^ are statistically about twice as large as those based on *F*, and *R*- factors based on ALL data will be even larger.

### Fractional atomic coordinates and isotropic or equivalent isotropic displacement parameters (Å^2^)

**Table:**

	*x*	*y*	*z*	*U*_iso_*/*U*_eq_	
O1	−0.00063 (12)	0.39282 (4)	0.01634 (8)	0.03761 (18)	
N1	0.15408 (11)	0.38503 (3)	0.13455 (8)	0.02345 (15)	
C1	0.31309 (14)	0.33197 (4)	0.11809 (9)	0.02439 (16)	
C2	0.48668 (14)	0.32849 (4)	0.26317 (9)	0.02460 (16)	
C3	0.48149 (12)	0.36207 (4)	0.39876 (9)	0.02137 (15)	
C4	0.29668 (13)	0.40897 (4)	0.41379 (9)	0.02327 (16)	
H4A	0.3526	0.4438	0.4887	0.028*	
H4B	0.1769	0.3865	0.4607	0.028*	
C5	0.20201 (12)	0.43844 (4)	0.25172 (8)	0.02002 (15)	
C6	0.64656 (13)	0.35531 (4)	0.53690 (10)	0.02476 (16)	
C7	0.77337 (15)	0.35377 (5)	0.65871 (11)	0.03106 (19)	
C8	0.42478 (17)	0.34218 (5)	−0.03471 (10)	0.0348 (2)	
H8A	0.5173	0.3808	−0.0220	0.052*	
H8B	0.3112	0.3474	−0.1280	0.052*	
H8C	0.5169	0.3048	−0.0515	0.052*	
C9	0.17967 (17)	0.26909 (4)	0.10502 (11)	0.0344 (2)	
H9A	0.1071	0.2635	0.2020	0.052*	
H9B	0.2793	0.2328	0.0947	0.052*	
H9C	0.0675	0.2709	0.0100	0.052*	
C10	−0.01285 (14)	0.47358 (5)	0.27003 (10)	0.02967 (18)	
H10A	−0.1216	0.4428	0.3008	0.045*	
H10B	−0.0704	0.4939	0.1674	0.045*	
H10C	0.0155	0.5066	0.3536	0.045*	
C11	0.36583 (13)	0.48486 (4)	0.18847 (10)	0.02556 (16)	
H11A	0.3027	0.5012	0.0826	0.038*	
H11B	0.5043	0.4622	0.1786	0.038*	
H11C	0.3953	0.5209	0.2637	0.038*	
H2A	0.607 (2)	0.2984 (7)	0.2539 (16)	0.042 (3)*	
H7A	0.870 (2)	0.3546 (8)	0.7563 (18)	0.057 (4)*	

### Atomic displacement parameters (Å^2^)

**Table:**

	*U*^11^	*U*^22^	*U*^33^	*U*^12^	*U*^13^	*U*^23^
O1	0.0374 (4)	0.0424 (4)	0.0274 (3)	0.0000 (3)	−0.0174 (3)	−0.0025 (3)
N1	0.0240 (3)	0.0261 (3)	0.0183 (3)	−0.0032 (2)	−0.0049 (2)	−0.0005 (2)
C1	0.0298 (4)	0.0241 (4)	0.0186 (3)	−0.0030 (3)	0.0005 (3)	−0.0028 (3)
C2	0.0266 (4)	0.0241 (4)	0.0223 (3)	0.0018 (3)	0.0000 (3)	−0.0008 (3)
C3	0.0221 (3)	0.0224 (3)	0.0187 (3)	0.0004 (2)	−0.0010 (2)	0.0020 (2)
C4	0.0242 (3)	0.0289 (4)	0.0158 (3)	0.0044 (3)	−0.0007 (3)	0.0006 (3)
C5	0.0189 (3)	0.0234 (3)	0.0167 (3)	−0.0001 (2)	−0.0017 (2)	0.0005 (2)
C6	0.0259 (4)	0.0242 (4)	0.0231 (3)	0.0030 (3)	−0.0008 (3)	0.0009 (3)
C7	0.0307 (4)	0.0341 (4)	0.0262 (4)	0.0056 (3)	−0.0049 (3)	0.0002 (3)
C8	0.0428 (5)	0.0408 (5)	0.0219 (4)	−0.0050 (4)	0.0080 (3)	−0.0045 (3)
C9	0.0450 (5)	0.0264 (4)	0.0303 (4)	−0.0095 (3)	−0.0009 (4)	−0.0039 (3)
C10	0.0227 (4)	0.0383 (5)	0.0273 (4)	0.0073 (3)	0.0006 (3)	0.0031 (3)
C11	0.0242 (3)	0.0241 (4)	0.0274 (4)	−0.0034 (3)	−0.0006 (3)	0.0026 (3)

### Geometric parameters (Å, °)

**Table:**

O1—N1	1.2858 (9)	C6—C7	1.1975 (12)
N1—C5	1.4854 (10)	C7—H7A	0.944 (15)
N1—C1	1.4874 (11)	C8—H8A	0.9800
C1—C2	1.5057 (11)	C8—H8B	0.9800
C1—C9	1.5368 (12)	C8—H8C	0.9800
C1—C8	1.5391 (12)	C9—H9A	0.9800
C2—C3	1.3360 (11)	C9—H9B	0.9800
C2—H2A	0.973 (13)	C9—H9C	0.9800
C3—C6	1.4381 (10)	C10—H10A	0.9800
C3—C4	1.5082 (11)	C10—H10B	0.9800
C4—C5	1.5314 (10)	C10—H10C	0.9800
C4—H4A	0.9900	C11—H11A	0.9800
C4—H4B	0.9900	C11—H11B	0.9800
C5—C10	1.5255 (11)	C11—H11C	0.9800
C5—C11	1.5322 (11)		
			
O1—N1—C5	118.39 (7)	C7—C6—C3	174.02 (9)
O1—N1—C1	116.41 (6)	C6—C7—H7A	177.0 (10)
C5—N1—C1	122.76 (6)	C1—C8—H8A	109.5
N1—C1—C2	111.10 (6)	C1—C8—H8B	109.5
N1—C1—C9	107.01 (7)	H8A—C8—H8B	109.5
C2—C1—C9	109.09 (7)	C1—C8—H8C	109.5
N1—C1—C8	109.79 (7)	H8A—C8—H8C	109.5
C2—C1—C8	109.60 (7)	H8B—C8—H8C	109.5
C9—C1—C8	110.22 (7)	C1—C9—H9A	109.5
C3—C2—C1	124.60 (7)	C1—C9—H9B	109.5
C3—C2—H2A	120.3 (8)	H9A—C9—H9B	109.5
C1—C2—H2A	115.1 (8)	C1—C9—H9C	109.5
C2—C3—C6	122.69 (7)	H9A—C9—H9C	109.5
C2—C3—C4	120.60 (7)	H9B—C9—H9C	109.5
C6—C3—C4	116.70 (7)	C5—C10—H10A	109.5
C3—C4—C5	112.67 (6)	C5—C10—H10B	109.5
C3—C4—H4A	109.1	H10A—C10—H10B	109.5
C5—C4—H4A	109.1	C5—C10—H10C	109.5
C3—C4—H4B	109.1	H10A—C10—H10C	109.5
C5—C4—H4B	109.1	H10B—C10—H10C	109.5
H4A—C4—H4B	107.8	C5—C11—H11A	109.5
N1—C5—C10	109.04 (6)	C5—C11—H11B	109.5
N1—C5—C4	107.72 (6)	H11A—C11—H11B	109.5
C10—C5—C4	109.47 (6)	C5—C11—H11C	109.5
N1—C5—C11	108.95 (6)	H11A—C11—H11C	109.5
C10—C5—C11	109.87 (7)	H11B—C11—H11C	109.5
C4—C5—C11	111.72 (6)		
			
O1—N1—C1—C2	179.37 (7)	C2—C3—C4—C5	−29.55 (11)
C5—N1—C1—C2	17.41 (10)	C6—C3—C4—C5	151.16 (7)
O1—N1—C1—C9	−61.65 (9)	O1—N1—C5—C10	33.57 (9)
C5—N1—C1—C9	136.40 (7)	C1—N1—C5—C10	−164.82 (7)
O1—N1—C1—C8	57.96 (9)	O1—N1—C5—C4	152.29 (7)
C5—N1—C1—C8	−103.99 (8)	C1—N1—C5—C4	−46.10 (9)
N1—C1—C2—C3	9.05 (11)	O1—N1—C5—C11	−86.35 (8)
C9—C1—C2—C3	−108.68 (9)	C1—N1—C5—C11	75.26 (8)
C8—C1—C2—C3	130.56 (9)	C3—C4—C5—N1	49.52 (8)
C1—C2—C3—C6	177.45 (7)	C3—C4—C5—C10	167.96 (7)
C1—C2—C3—C4	−1.79 (12)	C3—C4—C5—C11	−70.11 (9)

### Hydrogen-bond geometry (Å, °)

**Table:**

*D*—H···*A*	*D*—H	H···*A*	*D*···*A*	*D*—H···*A*
C7—H7A···O1^i^	0.944 (14)	2.354 (15)	3.2318 (13)	154.6 (13)

Symmetry codes: (i) *x*+1, *y*, *z*+1.
